# Why does network governance fail in managing post-disaster conditions in the Philippines?

**DOI:** 10.4102/jamba.v10i1.585

**Published:** 2018-11-12

**Authors:** Hazel D. Jovita, Achmad Nurmandi, Dyah Mutiarin, Eko P. Purnomo

**Affiliations:** 1 Department of Political Science, Iligan Institute of Technology of the Mindanao State University, Philippines; 2 Department of Government Affairs and Administration, Muhammadiyah University of Yogyakarta, Indonesia

## Abstract

Disasters are the litmus test of governance. The inherent complexity of disasters places government agencies and societies in vulnerable situations. This study uses the mixed-method approach to social network analysis in evaluating the network structure of the Philippine disaster management and its implications for disaster governance. A survey was conducted among the target 56 identified disaster response-related agencies and organisations from the disaster management networks of the most susceptible areas in the Philippines – Cities of Cagayan de Oro and Iligan, Province of Misamis Oriental and the overall Region 10 disaster response network, aimed at measuring the existing relationships among member agencies. Forty-four agencies and organisations were able to participate in the survey. Also, key informant interviews were conducted among the representatives of the lead agencies, non-government organisations and survivors of Typhoon Washi. The findings revealed that the mandated tall structure and the lead organisation form of network governance as stipulated in *Republic Act 10121* does not work in the regional and local disaster management networks in Region 10, particularly during Typhoon Washi in 2010. At the regional level, such structure does not build interdependencies among agencies, while at the local level, disaster response operations are constrained by bureaucratic protocols making disaster management networks ineffective. At the regional level, where many agencies and organisations are involved, the existing less centralised structure of decision-making should be transformed into a highly centralised structure, while disaster operations should be improved through coordination at all levels of disaster operations and intensified collaboration with non-government agencies.

## Introduction

Disasters naturally require decentralised decision-making and intensive human interactions (Kapucu & Van Wart 2008; Kirschenbaum 2004; Mileti 1999). Managing disasters involves dynamic processes that are ideal yet demanding. Thus, collaboration among organisations and government agencies is essential for the development of an effective strategy and better performance during disasters.

During disasters, government institutions are expected to have (Kapucu & Van Wart 2008):

the ability to assess and adapt capacity rapidly, restore or enhance disrupted or inadequate communications, utilise uncharacteristically flexible decision making, and expand coordination and trust of emergency response agencies despite the hurly-burly of the response and recovery efforts. (p. 280)

Aside from the fact that collaboration often occurs among proximal and like agencies (Simo & Bies 2007), collaborative disaster management faces various challenges which oftentimes lead to the failure of the response operations. Poor communication, inadequate planning, misguided and poorly executed leadership, and insufficient coordination with various stakeholders lead to collaborative failures (Kettl & Walters 2005; Menzel et al. 2006; Wise 2006).

The Philippines’ geographical location makes it susceptible to the most disastrous cyclones in the region and has experienced its own share of collaborative failures. In response to the devastating social disruptions caused by typhoons, institutional mechanisms, such as the *Republic Act 10121* (2009) or the Philippine law on disaster management, were implemented in 2010. The law aims to reduce the impacts of disasters in the country by adapting pro-active measures as well as cluster roach strategies among government agencies mandated to act upon disaster-related issues. The creation of the Disaster Risk Reduction Management (DRRM) Councils or networks at the national and local levels was aimed to guarantee the coordination and collaboration among government and non-government agencies in the implementation of programmes and policies stipulated in the disaster management plans of the country in order to provide an empowered and resilient Filipino communities. However, since the implementation of the law in 2010, the impacts of typhoons in the country are still enormous. Typhoons Washi in 2011, Bopha in 2012 and Haiyan in 2014 caused severe damage and unimaginable casualties. With this, it can be inferred that the implementation of the policy is poor, and the performance of the Philippine government is ineffective. This article explores the country’s disaster management governance structure and its implications for the performance of the disaster management network of Region 10 Philippines.

Jatmiko and Tandiarrang (2014), in their study of the Indonesian Maritime Agency, found that the existing structure of the agency does not support better communication among agencies which are crucial towards the agency’s performance. Meanwhile, Seng (2013) argued that the polycentric structure of Indonesian disaster management is ideal in responding to the cases of tsunamis in the country; however, it is not suitable to the norms of Indonesian political community. Moreover, Nurmandi et al. (2015) studied the different disasters in Indonesia and concluded that different governance structures are formed in each of the disasters they studied.

Thus, this article substantiates the existing works on the structural analysis of disaster governance networks and addresses the gap in the literature by utilising the mixed-method approach to social network analysis (SNA) in examining how the Philippine disaster management structure affects the disaster governance in the region. Disaster network characteristics and network centrality are particularly generated to analyse the network structure and its implications for disaster governance.

### Theoretical background

In studying public sector organisations, the whole network approach is widely utilised as the framework of analysis as it examines the connections that are both present and absent among a defined set of organisations, indicating the extent to which the organisations are working with one another to achieve a common goal (Provan & Lemaire 2012). Moreover, it examines the multilateral relations that define a whole network and that are essential for achieving a collective outcome.

Provan, Fish and Sydow (2007) suggested that whole networks should be analysed based on network governance, network leadership and management, and network performance. Network governance, which refers to the coordination mechanisms of a network that focus on the network as the unit of analysis in order to guide the network in a steady state (Provan & Kenis 2005), has three modes: shared or self-governance, which is characterised by the easy formation and high levels of commitment but with frequent meetings, lack of clear goals and difficulty in reaching consensus; lead organisation, which is depicted by the efficiency of clear network direction and management but faces the potential for lead organisation domination and low participation from the members; and network administrative organisation, which is illustrated as an entity that manages the network and comes with higher operation costs, a more complex administration process and a potential loss of control and decision authority for some network members. Meanwhile, Milward and Provan (2006) suggested that leadership and management follow the task framework that guides inter-organisational network leaders and managers in inter-organisational networks no matter what governance mode they choose.

On the other hand network performance, often referred to as network effectiveness, is defined by Provan and Kenis (2008) as ‘the attainment of positive network-level outcomes that could not normally achieve by individual organisational participants acting independently’. One of their propositions is (Provan & Kenis 2008):

that as trust becomes less densely distributed throughout the network, as the number of participants gets larger, as network goal consensus declines, and as the need for network-level competencies increases, brokered forms of network governance, like lead organisation and NAO, are likely to become more effective than shared-governance networks. (p. 237)

In the same vein, Raab, Manna and Cambre (2013) explored the way in which network structure, network context and network governance mode relate to network effectiveness. The results showed that the configurations for network effectiveness included low density and high centralisation as necessary conditions. Nevertheless, high density was a sufficient predictor of network effectiveness, while age, system stability and centralised integration are necessary but not sufficient conditions for effectiveness.

Ensuring that public value is fostered, as it is the ultimate goal of collaborative engagements, re-assessments and evaluation are made. Bardach (1998) posited that collaboration should only be valued if it creates better organisational performance. Hence, organisational or network performance should be assessed according to the targets attained by the network or the goal-achievement method (Agranoff 2007). Milward and Provan (2006) argued that the assessment of the performance of the network should be made by the community or stakeholders it is trying to serve. Meanwhile, Provan and Kenis (2008) and Raab et al. (2013) suggested that network structure and governance affect network effectiveness in such a way that, as the network has high density or too many members, trust is less distributed throughout the network, and goal consensus declines, resulting in less effective networks. Thus, a low density and a highly centralised network with a lead organisation are likely to characterise effective networks.

Broadly, networks are (O’Toole 1997):

structures of interdependence with many or multiple organisations made up many parts wherein one unit is not merely the formal subordinate of the others in some larger hierarchical arrangement. (p. 45)

The network structure is the most noticeable acreage of a network (Anklam 2007). Jatmiko and Tandiarang (2015) cited Anklam (2007) in explaining the patterns of network structures: centralised, mesh, hub-and-spoke, clusters, and core or periphery. Hence, the structure of the network provides a glimpse of how it is governed and predicts the possible output of the collaboration. Jung, Mazmanian and Tang (2009) posited that one of the main functions of managers is to build networks and employ effective strategies and mechanisms to ensure the sustainability and success of the network.

Bryson, Crosby and Stone (2006) postulated that structures among collaborative actions change and tend to be flexible because of the ambiguity of membership and complexity on local environments. Such ambiguity arises from many features of membership, including perceptions of who belongs to the collaboration and what these members actually represent. Moreover, the hierarchies of collaboration, in which individuals and organisations are often members of overlapping partnerships, further exacerbated the ambiguity of memberships. On the contrary, governance among networks determines the survival and success of the network or collaboration. Bryson et al. (2006) viewed governance, characterised by the initial agreement, leadership, planning, trust and managing conflict as a set of coordinating and monitoring activities that occur in the network for it to survive. Apparently, governance is highly dependent on the structure of the network, and as Bryson et al. (2006) emphasised, the choice of type of governance structure is likely to influence network effectiveness. The study of Bryson, Crosby and Stone (2015) suggested that agreements are attained if public managers adopt an inclusive process which is enabled by a flat structure.

On the contrary, Dalton and Upchurch (1978) explained that flat and tall structures pertain to the number of hierarchical levels of the organisation where the span of control for the tall structure is narrower, while the span of control for flat structure can be wider. They concluded that the organisation is structured in such a way that it fits its intended functions, and therefore, the structure of the organisation may vary but ‘they may remain within a reasonable range in which there will be no difference in performance attributable to structure’ (Dalton & Upchurch 1978).

Generally, this article utilises the whole network theory as its framework of analysis in examining the structure of the network based on its characteristics and centrality scores. For this research, the terms DRRM Council is used interchangeably with DRRM network or disaster management network. Also, network or governance structure refers to the structural configuration and characteristics of the networks, while network leadership and management pertains to network governance. Lastly, the term ‘dominance’ in the network refers to the agencies and organisations that may not be part of the mandated and authorised agencies to lead the disaster operations but are able to exercise leadership in the network.

### The Philippine disaster response during typhoons

Upon the enactment of the *Republic Act 10121* in 2010, all government units adapted the National DRRM Plan with specific modifications on its operations according to what is appropriate and applicable in the context and capacity of every community. Based on the level of implementation to disaster response, the Barangay DRRM Council will respond if a barangay is affected; if two or more barangays are affected, the City or Municipal DRRM Council will manage the operations, while the Provincial DRRM Council will respond if two or more cities or municipalities are affected. In cases where two or more provinces are affected, the Regional DRRM Council will direct the response operations, and if two or more regions are affected, the National DRRM Council (NDRRMC) will lead the disaster operations.

The NDRRMC structure, as well as its composition, is parallel among Local Government Units (LGUs): provinces, cities and municipalities. At the national level, the Department of National Defense (DND) heads the NDRRMC, and the Office of the Civil Defense (OCD) is the DND’s counterpart at the regional level, while among LGUs, their respective Local Chief Executives (LCEs) (Governor or Mayor) head the Council. DRRM Offices are established to assist the implementation of disaster-related activities agreed upon and approved by the DRRM Council composed of different agencies and non-government organisations. Generally, the DRRM Council is divided into four clusters or pillars of disaster management with lead agencies who serve as vice-chairs in the Council: the Department of Science and Technology (DOST) leads the Prevention and Mitigation Cluster; the Department of Interior and Local Government (DILG) leads the Preparedness Cluster; the Department of Social Welfare and Development (DSWD) heads the Response Cluster and the National Economic Development Authority (NEDA) heads the Rehabilitation and Recovery Cluster.

**FIGURE 1:**
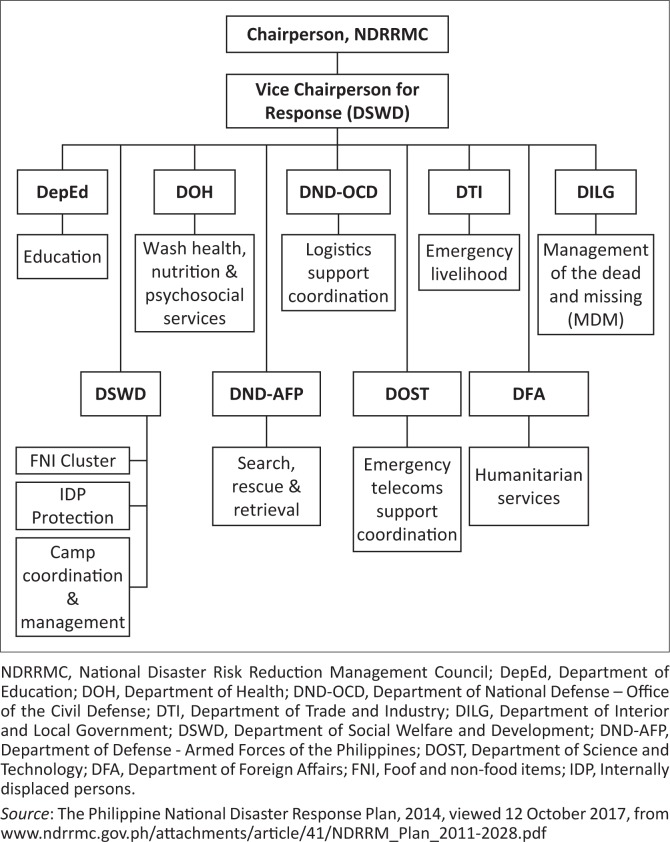
The organisational structure of the Philippine disaster response.

Moreover, to facilitate the response operations, the cluster approach was adopted. The response clusters are part of the NDRRMC’s strategic action in providing humanitarian assistance and disaster response services. These are organised groups of government agencies that are designated to undertake coordination functions at the strategic level to provide resource support for the tactical response. The response cluster is composed of nine clusters led by the DSWD, being the vice-chair on disaster response to the DRRM Council. The response clusters, led by different government agencies, are composed of education, health and psychosocial support, logistics support, emergency livelihood, management of the dead and missing, camp coordination and management, search and rescue, emergency telecommunications support and humanitarian services clusters (see Figure 1).

Generally, these disaster response procedures in the Philippines reflect a centralised approach to disaster management. The hierarchical relationships and the efficiency of clear network direction and management among organisations in the local and national government suggest that the Philippine disaster management has a large structure with a lead organisation form of governance (Provan & Kenis 2005).

In practice, disaster management in the Philippines faced severe damages and challenges on the implementation of *Republic Act 10121* in 2010. Rasquinho et al. (2013) found that the major problems of Typhoon Washi in 2011 were the unreliable communication systems and inefficient equipment and capacities for immediate response. The power outage in the region and the offices at the regional level, which were expected to facilitate the entire response operations, were affected by flood themselves; thus, it undermined their capacities to address the demand for operations (Rasquinho et al. 2013). Similarly, the impacts on the power outage and the lack of access to the affected areas were the main challenges after Typhoon Bopha left the country in 2012. The International Federation of Red Cross and Red Crescent Societies (2013) reported that the entire disaster response operations were delayed because the roads and bridges were impassable and water supply was disrupted in many areas. In the same vein, there was a lack of clarity on who will lead the disaster response operations after Typhoon Haiyan in 2013. Enriquez (2013) explained that the coordination of tasks was unclear, and there were:

shortages of tents and satellite phones in the first few days after the disaster, which the NDRRMC sourced from other agencies such as Public Works and Highways and the Philippine Navy. (p. 1)

## Research methods

This study uses the mixed-method approach to SNA in evaluating the network structure of Philippine disaster management and its implications for disaster governance. Social network analysis that provided the overview of the network’s topology in terms of its density, diameter and average distance metrics, and of the network’s centrality in terms of degree, betweenness and closeness centrality metrics, determined which organisation or agency held the central role within a network.

**FIGURE 2:**
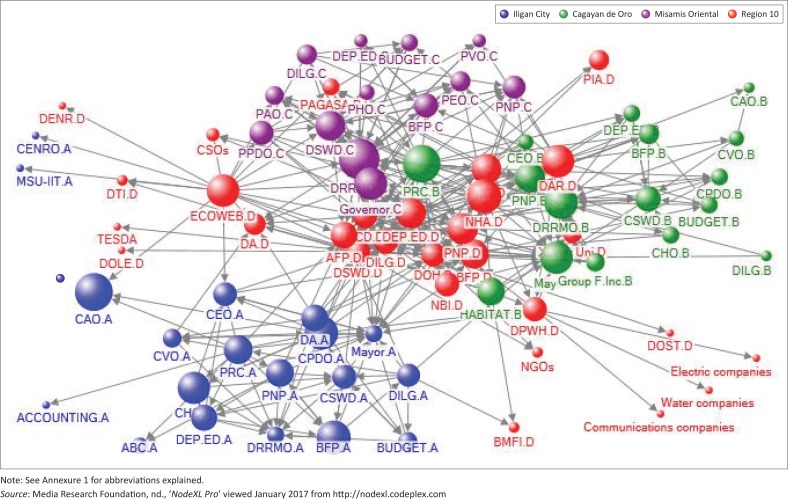
The overall Region 10 disaster risk reduction management network structure.

A survey was conducted among the target 56 identified disaster response-related agencies and organisations from the disaster management networks of the most susceptible areas in the Philippines – Cities of Cagayan de Oro and Iligan, Province of Misamis Oriental and the overall Region 10 disaster response network, aimed at measuring the existing relationships among member agencies. Forty-four agencies and organisations were able to participate in the survey: Region 10 – 7, Misamis Oriental – 11, Cagayan de Oro City – 14 and Iligan City – 11. Also, key informant interviews were conducted among the following sector: (1) representatives of the lead agencies (see Figure 1), (2) non-government organisations (Philippine Red Cross, Habitat Foundation, Church and Touch Foundation Incorporated) and (3) 10 survivors, on the basis of their involvement in the disaster management operations of the region.

The network’s topology and centrality measures are the main foundation for understanding the network and governance structure of Philippine disaster management. NodeXL software was used to process and analyse the data. Primarily, the topology of the network – density, diameter and average distance – was generated. The network’s density describes the portion of the potential connections with a network that are actual connections. A ‘potential connection’ is a connection that could potentially exist between two ‘nodes’ – regardless of whether or not it actually does. By contrast, an ‘actual connection’ is one that actually exists. To assess the density of the network, the following formula is applied: Total Possible Edges: # Nodes* *(# Nodes-1)/2; Density: Actual Edges/Possible Edges*. The result of the formula determines if the generated density of the network is considered low or high. Moreover, network diameter is the shortest distance between the two most distant nodes in the network. In other words, once the shortest path length from every node to all other nodes is calculated, the diameter is the longest of all the calculated path lengths. Meanwhile, average distance refers to the shortest path between nodes.

Centrality analysis gives a rough indication of the social power of a node based on how well they ‘connect’ to the network. A highly centralised network is dominated by one person who controls information flow. A less centralised network has no single point of failure. People can still pass on information even if some communication channels are blocked. The centrality of an entity is analysed using the network’s degree, betweenness and closeness measures. Degree centrality measures how connected an entity is by counting the number of direct links each entity has to others in the network; betweenness centrality measures the number of paths that pass through each entity, whereas closeness centrality measured the proximity of an entity to the other entities in the social network.

This study measured the characteristics of the disaster management networks in terms of high or low density, diameter and average distance. Also, the influence of each agency in their respective disaster management networks was assessed based on the centrality measures – degree, betweenness and closeness. Nodes, as referred to in this study, pertain to the disaster response-related agencies and organisations. Specifically, in Table 1, ‘node count’ refers to the actual relationships in the network, while ‘edges’ refer to the agencies and organisation mentioned by each node. Meanwhile, the term ‘lead agency’ or ‘agencies’ refers to the authorised and mandated agencies to lead the disaster-related operations in the country as stipulated in the Philippine law on disaster management or *Republic Act 10121*.

**TABLE 1:** Topographic metrics and centrality scores of the networks.

Variable	Formal authority network				
	Iligan	Cagayan de Oro	Misamis Oriental	Region 10	Overall
**Topographic metrics**					
Node count; Edges	27–71	39–77	26–45	58–172	78–313
Density	0.2193 > 0.2022 (high)	0.1079 > 0.1039 (high)	0.1692 > 0.1380 (high)	0.0526 < 0.10405 (low)	0.1165 < 0.1563 (low)
Diameter	4	5	2	4	5
Average distance	2.003	2.371	1.760	2.508	2.401
**Centralisation measures**					
Degree	5.704	4.103	4.231	5.586	9.091
Betweenness	14.037	27.231	10.385	88.517	54.442
Closeness	0.019	0.011	0.022	0.007	0.006

## Findings

The data showed interesting findings among the characteristics of the governance networks in the LGUs and at the regional level. Table 1 shows that the Iligan City DRRM network has a 0.219 density score, while Cagayan de Oro has 0.108, Misamis Oriental has 0.169 and Region 10 has a 0.05 density score. The value of the density scores suggests that in the LGUs of Misamis Oriental, Cagayan de Oro and Iligan City, there are a number of connections (high-density) among their respective networks. However, the density of the networks of the Region 10 DRRM Council and the overall DRRM network in the region is low, which implies fewer connections among member agencies in the network. This observation is plausible based on the frequency of the actual interaction among members of the network. When interviewed on 06 December 2016, Ms. A. Caneda explained that:

‘the Regional DRRM Council gathers at least 4 times in a year as it is required by Republic Act 10121 and during these regular meetings, some members are not able to attend due to their respective meetings and appointments while other agencies send their staff to represent them.’ (Head of the OCD, Ms. A. Caneda)

The comparison of the diameter metric scores reveals that the Misamis Oriental DRRM Council has a diameter of 2 nodes with an average distance of 1.76 nodes. On the contrary, Iligan, Cagayan de Oro, Region 10 and the overall network have a diameter of 4, 5, 4 and 5 nodes, with an average distance of 2.003, 2.371, 2.508 and 2.401 nodes, respectively. These data suggest that the local disaster management networks, primarily the Misamis Oriental DRRM network, have more connections than the rest of the disaster networks. Generally, the networks’ high-density scores, lower diameter and lower average distance are products of a smaller number of network members, which suggest easier familiarity between and among agencies. On the contrary, the regional and overall disaster management networks’ low density and higher average distance suggest a less connected relationship between member agencies. However, the characteristics of the local disaster management networks show that there is high density and higher diameter and the average distance is almost the same as the rest of the networks.

Therefore, member agencies in the regional and local disaster management networks are sparsely connected as revealed in the diameter and average distance scores. However, in the local disaster networks, more connections are established among member agencies as implied by the higher density scores. This situation is validated in the statement of the Iligan City Social Welfare and Development focal person, Ms. P. Mantos. When interviewed on 25 November 2016, she stated that:

‘I can say that I am already familiar with the focal person of the different agencies in the City DRRM Council, except for the new ones. We have been seeing each other in the different activities of the City. However, our only real interaction is only during the regular meeting/s of the Council, disaster management planning or response. Some of these focal persons are replaced by their agencies and are assigned to another unit. It was quite challenging to request any information or data with them during disaster planning, especially during disaster response operations.’ (City Social Welfare and Development focal person, Ms. P. Mantos)

Furthermore, these findings suggest that there are processes in the network that generate minimal information sharing which causes ineffective coordination and inefficient disaster response operations in Region 10.

The centralisation scores of the four networks revealed that in terms of degree centrality, the Region 10 DRRM Council has the least degree centrality, while the DRRM Council of the LGUs of Misamis Oriental, Cagayan de Oro and Iligan has high degree centrality. The degree centrality score of the overall DRRM network in the Region is 9.091 degree relationship, which is lower considering the number of its member agencies. Hence, the networks with the smaller number of member agencies and organisations are relatively highly centralised. Members who are mandated by the law to take part in the disaster management activities have higher degree centrality scores than those members whose membership is on a voluntary basis. Thus, the disaster management network at the regional level is less centralised, while the local management networks are highly centralised in terms of degree centralisation, particularly the Misamis Oriental disaster management network. The less centralised character of the regional network is confirmed by the statement of the Region 10 DILG focal person, Ms H. Ocena. When interviewed on 11 December 2016, she stated that:

‘in the Regional level, each cluster in disaster response is led by a certain agency. For example, the Department of Education leads the Education Cluster, Department of Health for the Health Cluster. The OCD oversees these operations. However, I cannot say that the OCD or a certain agency in Region 10 is most influential or more dominant in the network.’ (Region 10 DILG focal person, Ms H. Ocena)

Meanwhile, in terms of betweenness, the overall network and the Region 10 DRRM network have high betweenness centralisation scores with 54.442 and 88.517 degree relationships, respectively (see Table 1), while the betweenness centralisation scores of Iligan, Cagayan and Misamis Oriental – 14.037, 27.231 and 10.385 degree relationships – are relatively low. These data suggest that the regional offices work as a bridge in the entire network in terms of sharing information and resources during disaster-related operations (see Figure 2). Hence, the regional agencies control the flow of information in the network. Moreover, the Region 10 disaster network may have fewer connections, yet these are significant ties which are vital for the network operations as revealed by its betweenness centralisation score (see Table 1). Additionally, the closeness centrality scores of DRRM networks in Region 10 (see Table 2) suggest the proximity of the regional agencies to the other agencies and organisations in the entire regional network. This validates the finding that the regional disaster management network is highly centralised, while the local disaster management networks are less centralised in terms of betweenness. The highly centralised nature of the regional network in terms of betweenness is validated in the statement of the Region 10 DSWD focal person, Ms E. Cardona. When interviewed on 15 November 2016, she mentioned that:

**TABLE 2:** The summary of the centrality scores of each disaster risk reduction management network.

Variable	Degree	Score	Betweenness	Score	Closeness	Score
**Centrality scores of the Iligan City DRRM network**						
1	CPDO.A	14	CPDO.A	84.355	PRC.A	0.026
2	PRC.A	14	PRC.A	82.701	CPDO.A	0.025
3	DA. A	12	DILG.A	54.058	Mayor.A	0.024
4	CSWD.A	11	DA. A	42.192	CSWD.A	0.024
5	Mayor. A	11	CSWD.A	26.220	DRRMO.A	0.023
6	DRRMO.A	10	Mayor. A	24.685	DA.A	0.022
7	DILG.A	9	CEO.A	19.553	CEO.A	0.022
8	CEO.A	8	DRRMO.A	12.911	CHO.A	0.021
9	DEP.ED. A	8	CVO.A	10.938	DEP.ED.A	0.021
10	PNP.A	8	PNP.A	7.985	CVO.A	0.021
**Centrality scores of the Misamis Oriental DRRM network**						
1	DRRMO.C	25	DRRMO.C	235.233	DRRMO.C	0.040
2	DSWD.C	12	DSWD.C	17.233	DSWD.C	0.026
3	PPDO.C	9	PPDO.C	8.617	PPDO.C	0.024
4	BFP.C	8	BFP.C	5.117	BFP.C	0.024
5	DILG.C	7	DILG.C	1.700	DILG.C	0.023
6	Governor.C	6	PHO.C	1.500	Governor.C	0.023
7	PEO.C	5	Governor.C	0.400	PEO.C	0.022
8	PHO.C	4	PEO.C	0.200	PHO.C	0.022
9	PNP.C	4	PNP.C	0.000	PNP.C	0.022
10	BUDGET.C	4	AFP.D	0.000	BUDGET.C	0.022
**Centrality scores of the Cagayan de Oro City DRRM network**						
1	PRC.B	21	PRC.B	305.738	PRC.B	0.016
2	DRRMO.B	16	DRRMO.B	191.487	DRRMO.B	0.016
3	PNP.B	12	HABITAT.B	142.204	PNP.B	0.014
4	HABITAT.B	12	PNP.B	76.942	DSWD.D	0.014
5	CSWD.B	10	Touch F. Inc.B	74.069	DILG.D	0.013
6	Mayor.B	7	DSWD.D	52.929	Mayor.B	0.013
7	DSWD.D	6	Mayor.B	48.223	OCD.D	0.013
8	Touch F. Inc.B	6	CVO.B	37.000	CSWD.B	0.013
9	BFP.B	5	CSWD.B	33.417	BFP.B	0.012
10	CHO.B	5	DILG.D	24.956	HABITAT.B	0.012
**Centrality scores of the Region 10 DRRM network**						
1	DRRMO.C	28	DSWD.D	347.327	Accounting. A	0.004
2	DSWD.D	25	ECOWEB.D	343.293	ABC.A	0.004
3	PRC.B	24	AFP.D	275.800	Electric co.	0.004
4	DILG.D	23	DRRMO.C	252.923	water co.	0.004
5	DOH.D	22	DPWH.D	239.017	DRRMO.B	0.004
6	OCD.D	20	Mayor.A	205.844	DENR.D	0.004
7	AFP.D	20	DOH.D	203.066	MSU-IIT.A	0.004
8	NHA.D	20	DAR.D	201.270	CENRO.A	0.004
9	Mayor.A	19	PRC.A	165.561	CAO.B	0.004
10	DRRMO.B	19	NHA.D	161.394	DEP.ED. A	0.005

Note: ‘Degree’, ‘Betweenness’ and ‘Closeness’ centrality are aimed to measure the influence of the institutions involved in disaster response management. Degree centrality determines the institution’s connectedness or popularity; while betweenness centrality determines the institutions who are in the communication paths of the network. Meanwhile, closeness centrality determines the reach of one institution to the rest of the network and suggests how fast an institution can connect to the network.

‘Scores’ show the centrality measurements for degree, betweenness and closeness. By identifying the number of links that lead into and out of the node/agency, scores for degree centrality determines the most influential institution, while the scores in betweenness centrality, which quantifies the number of times a node acts as a bridge along the shortest path between two other agencies, suggest the vital role of the agency in the network as many other institutions are connected by that agency. On the other hand, the scores in closeness centrality, which are generated by calculating the mean length of all the shortest paths from an agency to all other agencies in the network, shows which network has the highest or easiest access to the rest of the network. Therefore, the higher the scores in these centrality measures, the more influential the agency is.

See Annexure 1 for abbreviations explained.

DRRM, disaster risk reduction management.

‘disaster management in the region is clustered to properly address the specific needs and concern during disaster response. Each cluster has its own lead agency. As we take over the disaster operations from the LGUs, we always make sure that we provide the needs of the affected communities by facilitating the processes involved particularly by coordinating with other agencies and organisations across the Region.’ (Region 10 DSWD focal person, Ms E. Cardona)

Table 2 shows the list of agencies from each network with the highest scores according to the centrality measures: degree, betweenness and closeness. Table 2 reveal that the dominant agencies in the DRRM networks of Iligan and Cagayan de Oro are not exactly the agencies who belong in the mandated structure of the National Disaster Response Plan (Figure 1). Interestingly, the involvement of the non-government agencies such as the Philippine Red Cross, Habitat Foundation and Group Foundation Incorporated implies that disaster response-related activities in the LGU could be improved and sustained. Hence, such collaboration needs to be strengthened. When interviewed on 06 January 2017, the representative of the Philippine Red Cross in Iligan City, Mr. G. Galucan, shared that ‘a day after Typhoon Washi hit the Region, we immediately mobilised our volunteers and resources to help in the rescue operations’. The Touch Foundation Incorporated focal person recounted their experience too. When interviewed on 10 January 2017, Mr I. Borja narrated that:

‘our organisation was not ready for disaster response, but we were receiving donations from our partners from all over the Philippines, so we attempted to coordinate with the LGU, but there was no focal person in charge to receive the donations. Worst, at that time, the City Mayor of Cagayan de Oro was not around, and the impact of Washi overwhelmed the DSWD, DRRM Office and other agencies in the City. So, we capacitated ourselves, mobilised our members and distributed the goods to the victims of the typhoon in coordination with Xavier University and Catholic Church of Cagayan de Oro.’ (The Touch Foundation Incorporated focal person, Mr I. Borja)

The absence of bureaucratic protocols in the operations of the mentioned non-government agencies is one of the factors that enable them to respond faster and effectively. Noteworthy, the characteristics of the disaster management networks described above – highly dense with sparsely connected member agencies and less centralised in terms of degree – imply that the existing structure of the disaster management networks is not suitable in Region 10 as manifested by its ineffective disaster response operations. When interviewed on 13 November 2016, most of the interviewed survivors of Typhoon Washi shared:

‘we were not rescued in our homes. We brought ourselves to the evacuation centers near us, bringing nothing but ourselves and family members. We had nothing, and we were not able to contact our relatives because there was no electricity and no signal on cellular phones. We relied on the relief goods distributed by NGOs and private agencies. Relief goods from the government were delivered weeks after the typhoon.’

The Iligan City former DRRM Officer, Mr A. Bendijo, expounded that bureaucratic protocols did not work in their favour. When interviewed on 28 November 2016, Mr A. Bendijo said that:

‘the government has funds for disaster response but prior to its utilisation, the Local DRRM Council has to convene first and declared that the City is in the State of Calamity, and it took weeks for both DRRM Councils to convene. Also, the funds are subjected to the regular procurement processes of the government. So, it took a while for the City Government to utilise the fund and fully address the needs of the Typhoon survivors.’ (Former Iligan City DRRM Officer, Mr A. Bendijo)

## Discussion

Significant findings are observed from the chosen DRRM networks in the Philippines. Primarily, in terms of the topographic characteristics of the networks, the density scores are high among the LGUs of Misamis Oriental, Cagayan de Oro and Iligan City, while there is a low density in the Region 10 DRRM Council. These data suggest that the smaller the number of network members, the higher the connections between and among member agencies and organisations are established. Similarly, in networks with more members, such as Region 10, connections are hardly established because agencies and organisations are divided among clusters.

Networks with low density imply fewer connections among member agencies. Fewer connections further imply that there are fewer cases or opportunities for face-to-face encounters or similar activities that enhance the quality of the relationship between and among agencies, which lead to fewer interdependences and low trust in the network (Ansell & Gash 2008). Kapucu (2005) noted that effective response and recovery operations require collaborations and trust between government agencies at all levels and between the public and non-profit sectors. Providing incentives fosters inter-organisational communication and trust that enables accelerating inter-organisational network coordination in emergency management response operations (Ansell & Gash 2008; Kapucu 2006; Tang & Tang 2014). Building interdependencies among agencies and organisations through interactive processes increases trust, builds social capital and can develop into collaborative culture which can substantially increase the speed of decision-making and can lead to successful collaborations (Ansell & Gash 2008; Emerson, Nabatchi & Balogh 2012; Jung, Mazmanian & Tang 2009; Kapucu, Arslan & Demiroz 2010; Paraskevopoulos 2010; Shaw & Goda 2004; Shimada 2015). Meanwhile, networks with low density and highly centralised nature are effective conditions for network effectiveness (Raab et al. 2013). However, the regional disaster management network has low density and is less centralised in terms of degree, which suggests weak disaster management structure as characterised by low trust, lack of interdependencies and slow-paced decision-making during disaster management operations in Region 10.

Meanwhile, the presence of the cluster-based lead agency in the region is shown in the centralisation scores of the network (see Table 2). Table 2 shows that in the regional disaster network, the agencies with the most number of connections are considered to be the most important agencies in the network, similar to the agencies mandated by the *Republic Act 10121* to lead the disaster response operations in the region according to their respective clusters. Thus, in terms of centralisation scores, the entire disaster management network in Region 10 mirrors the mandated structure in the *Republic Act 10121*. According to the *Republic Act 10121*, the disaster management network is structured with a lead agency governing the activities in every cluster and is facilitated by the OCD. In terms of Region 10’s network characteristics, there are only 52 connections out of the 152 mentioned agencies. Hence, network density at the regional level is low. This implies that there is weak collaboration in the regional network, which resulted in the minimal information sharing and less effective and less efficient operations. Consequently, trust and interdependencies were low because of the lack of opportunities to have face-to-face encounters among member agencies at the regional level. These findings confirm the finding of Bharosa et al. (2008) that most agencies in collaborative efforts appreciate the advantages of collaboration but only a few are actually willing to collaborate.

Many scholars believe that disaster management networks should be decentralised. However, Kapucu (2006) argued that decision-making should be centralised to provide clear direction for disaster operations, which should be decentralised. Figure 2 suggests that in the regional network, no single agency leads the entire disaster response operations despite the implementation of the cluster approach. This finding is consistent with the observation during the 2014 Typhoon Haiyan response efforts ‘there was a lack of clarity on who was in charge from the national government’ (Enriquez, 2013). This finding suggests weak collaboration in the overall network of disaster management which resulted in minimal information sharing and less effective and less efficient operations. The minimal network information sharing supports the findings of Jatmiko and Tandiarrang (2014) that new structure should be built in order for information sharing in the network to be strengthened. These findings confirm the theory of Provan and Kenis (2008) that as the network members increases, the network should be governed by a lead-organisation in order to effectively coordinate network activities and decisions.

On the contrary, the disaster management networks in LGUs revealed distinctive findings. The LGUs of Misamis Oriental, Cagayan de Oro and Iligan City have high-density scores with relatively higher average distance and diameter. With a smaller number of member agencies, more connections within local disaster management networks are established.

Moreover, Table 2 revealed that the agencies involved in the ‘actual’ local disaster networks are different from the ‘mandated agencies’ according to the *Republic Act 10121* (see Figure 1). According to the actual governance structure (see Figure 2), there is no single agency that dominates their respective disaster response networks, except in the case of the Province of Misamis Oriental, where the role of the Provincial DRRM Office is glaring, being the biggest node in the provincial disaster network (followed by the Provincial DSWD). For the Cities of Iligan and Cagayan de Oro, the disaster networks are governed not just by the designated agencies of the LCE but also by non-government organisations such as the Philippine Red Cross, which acted voluntarily. The latter is not part of the mandated agencies but turned out to be influential and able to exercise leadership during the networks’ disaster response.

Furthermore, the tall structure and the lead organisation form of network governance (Provan & Kenis 2008), which is centralised in nature, do not work in the local disaster management networks because of the dominance of non-government agencies. This finding supports the conclusion of Lester and Krejci (2007) that leadership during disaster management is not about who holds the authority to lead and direct the disaster operations but, more importantly, about who exercises actual leadership in times of crisis. This study strengthens the theory of Bryson et al. (2006) that the ambiguity of membership, which lies in the hierarchy of collaboration where members have overlapping partnerships across networks, and complexity in local environments such as lack of implementation on existing (environmental) policies (Almarez, Penaroya & Rubio 2015) alter the structures among collaborative actions. On the contrary, ambiguity and complexity in local environments are simplified when agreements are attained through an inclusive collaborative process in the network, which is often achieved in a flat-structured organisation, instead of hierarchies (Bryson et al. 2015).

This study further corroborates the findings of Seng (2013) that the structure may be ideal, but it does not necessarily imply that it is suitable in the community as factors such as social norms and political culture might get in the way. This finding also confirms the conclusion of Kapucu and Van Wart (2008) that decentralised decision-making in the form of an excessive reliance on centralised authorities could bring more harm than good, particularly if the authorities are not fully committed to addressing needs and resolving the various challenges along the way. In the case of the LGU in the Philippines, the LCE holds the authority and serves as the emergency manager as mandated by the *Republic Act 10121*. Hence, emergency managers should fully grasp the value of collaboration by capacitating the members of the network and the community (Kapucu, Arslan & Collins 2010).

Therefore, this study suggests that a highly centralised disaster network with a shared governance and a flat structure should be considered to enhance the competence of the local agencies through an inclusive collaborative process in order to attain agreements, foster interdependencies and sustain reliable partnerships in the region’s disaster management networks. The strong presence of non-government agencies (Table 2) suggests that sustainable partnership or collaboration between non-government and government agencies could lead to a more effective disaster management network, thus better disaster response. With definite and sound government structures, civil society organisations can harness their potential in crisis situations which could go beyond rapid damage assessments (Alegado 2014; Paramita 2012). Thus, adopting a highly centralised network with shared governance in structuring the disaster management networks leads to sustainable and effective structures and processes in the disaster management operations.

### Limitations

One of the major limitations of this study is its scope. Disaster governance processes, as well as the degree of social capital among the affected communities using a stakeholder analysis, could be explored.

## Conclusion

Generally, the Philippine disaster management networks in Region 10 failed to respond effectively during Typhoon Washi in 2010 because of significant reasons. Primarily, the overall disaster management network in Region 10 has a low density, which means that trust and interdependencies were low because of the lack of opportunities to have face-to-face encounters among member agencies at the regional level. Moreover, decision-making in the regional disaster management network is less centralised, as revealed by the lack of a dominant or lead agency in the entire regional disaster management. This implies that there is weak collaboration in the regional network, which resulted in minimal information sharing and ineffective disaster response.

On the contrary, the disaster management networks in LGUs have high-density scores with relatively lower average distance and diameter because of the smaller number of member agencies. Hence, the smaller the network, the higher the connection. Despite the relative cohesiveness in the local management networks, the presence and dominance of non-government agencies imply lack of capacities in terms of decision-making and resources among the mandated agencies. With this limitations, the local network structures nurtures the existing bureaucratic protocols which make it more difficult for the local government units to operate effectively. These network characteristics reduce the capacities of the local disaster management networks, which leads to weak disaster operations.

Therefore, the tall structure and the lead organisation form of network governance (Provan & Kenis 2008), which is centralised in nature, do not work in the local and regional disaster management networks in Region 10, Philippines, because at the regional level, such structure does not build interdependencies among agencies, while at the local level, disaster response operations are constrained by bureaucratic protocols, which make disaster management networks less effective. Hence, shared governance should be explored. Structurally, a mixture of the forms of network governance – lead organisation and shared governance – should be investigated. At the national and regional levels, where many organisations are part of the network, centralised decision-making is necessary and disaster operations should be decentralised (Kapucu 2005). However, trust and inter-dependency should be cultivated in centralised networks to come up with effective mechanisms during disasters.

## Annexure 1

**TABLE 1-A1:** List of abbreviations defined.

Abbreviation	Description
CPDO.A	Iligan City City Development and Planning Office
PRC.A	Iligan City Philippine Red Cross
DA. A	Iligan City Department of Agriculture
CSWD.A	Iligan City City Social Welfare and Development
Mayor. A	Iligan City Office of the Mayor
DRRMO.A	Iligan City Disaster Risk Reduction and Management Office
DILG.A	Iligan City Deparmtent of Interior and Local Government
CEO.A	Iligan City City Engineer’s Office
DEP.ED. A	Iligan City Deparment of Education
PNP.A	Iligan City Philippine National Police
DRRMO.C	Misamis Oriental Provincial Disaster Risk Reduction and Management Office
DSWD.C	Misamis Oriental Provincial Social Welfare Department
PPDO.C	Misamis Oriental Provincial Planning and Development Office
BFP.C	Misamis Oriental Bureau of Fire Protection
DILG.C	Misamis Oriental Department of Interior and Local Government
Governor.C	Misamis Oriental Office of the Governor
PEO.C	Misamis Oriental Provincial Engineer’s Office
PHO.C	Misamis Oreintal Provincial Health Office
PNP.C	Misamis Oriental Philippine National Police
BUDGET.C	Misamis Oriental Budget Office
PRC.B	Cagayan de Oro City Philippine Red Cross
DRRMO.B	Cagayan de OroCity Disaster Risk Reduction and Management Office
PNP.B	Cagayan de Oro City Philippine National Police
HABITAT.B	Habitat for Humanity
CSWD.B	Cagayan de Oro City Social Welfare and Development
Mayor.B	Cagayan de Oro City Office of the Mayor
DSWD.D	Region 10 Department of Social Welfare and Department
Touch F. Inc.B	TOUCH Foundation Incorporated
BFP.B	Cagayan de Oro City Bureau of Fire Protection
CHO.B	Cagayan de Oro City Health Office
DILG.D	Region 10 Department of Interior and Local Government
DOH.D	Region 10 Department of Health
OCD.D	Region 10 Office of the Civil Defense
AFP.D	Region 10 Armed Forces of the Philippines
NHA.D	Region 10 National Housing Authority
Mayor.A	Iligan City Office of the Mayor
CVO.A	Iigan City Veterinary Office
CVO.B	Cagayan de Oro City Veterinary Office
ECOWEB.D	Ecosystems Work for Essential Benefits (EcoWEB)
DPWH.D	Region 10 Department of Public Works and Highways
DAR.D	Region 10 Department of Agrarian Reform
DA.A	Iligan City Department of Agriculture
CHO.A	Iligan City Health Office
DEP.ED.A	Iligan City Deparment of Education
Accounting. A	Iligan City Accounting Office
ABC.A	Iligan City Association of Barangay Captains
Electric co.	Electric Cooperative
water co.	Water Cooperative
DENR.D	Region 10 Department of Environment and Natural Resources
MSU-IIT.A	Mindanao State University - Iligan Institute of Technology
CENRO.A	Iligan City Environment and Natural Resources Office
CAO.B	Cagayan de Oro Agriculture Office

Note: Abbreviation listed in this table refer to abbreviation used in Figure 2 and Table 2.
